# Ayahuasca enhances creative divergent thinking while decreasing conventional convergent thinking

**DOI:** 10.1007/s00213-016-4377-8

**Published:** 2016-07-19

**Authors:** K. P. C. Kuypers, J. Riba, M. de la Fuente Revenga, S. Barker, E. L. Theunissen, J. G. Ramaekers

**Affiliations:** 1 Department of Neuropsychology and Psychopharmacology, Faculty of Psychology and Neuroscience, Maastricht University, Maastricht, The Netherlands; 2 Human Experimental Neuropsychopharmacology, Sant Pau Institute of Biomedical Research, Barcelona, Spain; 3 Department of Comparative Biomedical Sciences, Louisiana State University, Baton Rouge, LA USA

**Keywords:** Ayahuasca, Field study, Divergent creative thinking, Conventional convergent thinking, Creativity

## Abstract

**Introduction:**

Ayahuasca is a South American psychotropic plant tea traditionally used in Amazonian shamanism. The tea contains the psychedelic 5-HT_2A_ receptor agonist *N*,*N*-dimethyltryptamine (DMT), plus β-carboline alkaloids with monoamine oxidase-inhibiting properties. Increasing evidence from anecdotal reports and open-label studies indicates that ayahuasca may have therapeutic effects in treatment of substance use disorders and depression. A recent study on the psychological effects of ayahuasca found that the tea reduces judgmental processing and inner reactivity, classic goals of mindfulness psychotherapy. Another psychological facet that could potentially be targeted by ayahuasca is creative divergent thinking. This mode of thinking can enhance and strengthen psychological flexibility by allowing individuals to generate new and effective cognitive, emotional, and behavioral strategies. The present study aimed to assess the potential effects of ayahuasca on creative thinking.

**Methods:**

We visited two spiritual ayahuasca workshops and invited participants to conduct creativity tests before and during the acute effects of ayahuasca. In total, 26 participants consented. Creativity tests included the “pattern/line meanings test” (PLMT) and the “picture concept test” (PCT), both assessing divergent thinking and the latter also assessing convergent thinking.

**Results:**

While no significant effects were found for the PLMT, ayahuasca intake significantly modified divergent and convergent thinking as measured by the PCT. While convergent thinking decreased after intake, divergent thinking increased.

**Conclusions:**

The present data indicate that ayahuasca enhances creative divergent thinking. They suggest that ayahuasca increases psychological flexibility, which may facilitate psychotherapeutic interventions and support clinical trial initiatives.

## Introduction

Ayahuasca is a South American psychotropic plant tea used around the world for curing, divination, and to get in contact with another supernatural dimension. It contains the serotonergic 2A receptor agonist *N*,*N*-dimethyltryptamine (DMT) and monoamine oxidase inhibitors that render DMT orally active (McKenna 2004; Riba et al. 2006; Schenberg et al. 2015). Acute subjective effects usually start between 45 and 60 min post-administration and include visionary experiences with more intense emotions and increased rates of thinking when the eyes are closed (Riba et al. 2001, 2003). Working memory impairment after ayahuasca intake was shown to be limited to less experienced users while absent in more experienced users (Bouso et al. 2013). It was suggested that the latter group has developed mechanisms to compensate for the acute impairing effects of ayahuasca (Bouso et al. 2013). A study into the long-term effects found no evidence for detrimental effects on psychological or mental health, or cognitive abilities, in a 1-year follow-up study, compared to controls (Bouso et al. 2015).

Psychedelic drugs have gained a renewed interest as potential tools in therapy (Domínguez-Clavé et al. 2016). Double-blind studies on the acute and long-term effects of psilocybin on psychological function suggest that a psychedelic session can produce life-altering experiences and long-term improvements in personal well-being and induce a positive behavior change (e.g., Gouzoulis-Mayfrank et al. 1999a, b; Griffiths et al. 2006; Hasler et al. 2004). A majority of participants in a study of Griffiths and coworkers (2006) reported that the session was one of the most spiritually significant experiences of their lives (Griffiths et al. 2006). More recently, the National Survey on Drug Use and Health in the USA revealed that lifetime classic psychedelic use was associated with a significant reduction in psychological distress and suicidal thinking (Hendricks et al. 2015). It has been suggested that psychedelics such as ayahuasca might be, within an appropriate psychotherapeutic setting, useful in the treatment of substance use disorders because it can result in positive and lasting lifestyle changes (dos Santos et al. 2016). A recent study showed that ayahuasca enhances mindfulness-related capacities, a classic goal of certain psychotherapeutic interventions (Soler et al. 2015). Changes in behavior and lifestyle may also result from a psychedelic’s capacity to stimulate creative, divergent thinking, which has also been shown to be an important aspect in cognitive therapy (Forgeard and Elstein 2014). Divergent thinking is taken to represent a style of thinking that allows many new ideas being generated, in a context where more than one solution is correct. The best example is probably a brainstorming session, which has the aim of generating as many ideas on a particular issue as possible. In contrast, convergent thinking is considered a process of generating a single optimal solution to a particular problem. The latter emphasizes speed and relies on high accuracy and logic. Both divergent and convergent thinking are seen as two main ingredients of most creative activities even though other processes may also contribute (Guilford 1967; Wallas 1926).

Creative divergent thinking can enhance and strengthen psychological flexibility by allowing individuals to generate new and effective cognitive, emotional, and behavioral strategies on their own which helps them to adopt adaptive interpretations and coping styles (Forgeard and Elstein 2014). Interestingly, it was shown that acute administration of ayahuasca causes a decrement in the functional connectivity in parts of the default mode network (Palhano-Fontes et al. 2015). It was suggested that as a result, there is more cognitive flexibility and consequently potential enhanced creative thinking (Carhart-Harris et al. 2014). Interestingly, the acute effects of ayahuasca on creativity may also outlast the acute intoxication phase. Frecska et al. (2012) showed that creative thinking, especially originality, was increased 2 days after the end of a 2-week ayahuasca ceremony, when the acute intoxication subsided (Frecska et al. 2012).

The present study aimed to assess creative thinking in participants of ayahuasca sessions in a quasi-experimental study. Assessments of creative divergent thinking, as well as conventional convergent thinking, were scheduled prior to and during the acute effects of ayahuasca in two separate groups involving two different settings.

## Methods

### Participants

The investigators visited two spiritual ayahuasca-using groups and invited participants to enter the current study. Two different groups were approached to test whether potential effects of ayahuasca on creativity occur independently of setting or participant characteristics. A total of 26 participants consented after the goals and methods of the study were explained. Exclusion criteria included current psychiatric disorder and alcohol or other substance use disorders and evidence of current significant medical illness. All participants were Caucasian individuals of European and American (North and South American) and middle-Eastern descent. They all had an interest in psychoactive drugs for personal experimentation, and their reported motivation for ayahuasca use was to enhance introspection, self-knowledge, and personal growth.

Group 1 included 15 participants (10 women) with a mean ± SD age of 37.4 ± 5.8 and 15.5 ± 3.2 years of education. They all had prior experience with ayahuasca, having taken it on an average of 27.5 ± 33.4 occasions. Group 2 included 11 participants (7 women) with a mean ± SD age of 52.0 ± 13.0 and 18.4 ± 1.5 years of education. All had also prior experience with ayahuasca, having taken it on an average of 103.6 ± 152.9 occasions.

The study was conducted in accordance with the Declaration of Helsinki and subsequent amendments concerning research in humans and was approved by the Sant Pau Hospital Ethics Committee. All volunteers gave their written informed consent to participate. All participants had abstained from ayahuasca, other psychotropic substances, alcohol, and medications for at least 2 days before the assessment session.

### Study procedure

The assessment of each participant group was conducted separately. The setting in which ayahuasca was taken was quite similar in both ayahuasca groups. Ayahuasca was taken in a non-religious setting, and participants were not affiliated to any ayahuasca religion. The main motivation of the participants was to use ayahuasca as a tool for self-knowledge and introspection. Participants remained in dimly lit rooms, and recorded music was played throughout the sessions. Sessions were held in the evening, and in both groups, an initial ayahuasca dose was served and approximately 1 h after this initial dose, a second one was offered that the participants could take or not.

Creativity tests were administered twice: the pre-ayahuasca assessment was conducted in the afternoon around 3 h before the start of the session. The post-ayahuasca assessment was conducted during the acute inebriation around 1.5–2 h after the initial dose, after which the second dose was offered. The total ayahuasca volume taken by each participant was recorded, and samples (10 ml) of the ayahuasca teas were taken in both groups to determine the concentrations of DMT, harmine, tetrahydroharmine, and harmaline afterward. Alkaloid concentrations were determined using a previously described method and conducted by one of the researchers (SB) using a previously described method implementing liquid chromatography-electrospray ionization-tandem mass spectrometry (McIlhenny et al. 2009).

### Ayahuasca

The ayahuasca tea used by group 1 contained the following alkaloid concentrations: 0.65 mg/ml DMT, 0.84 mg/ml harmine, 0.76 mg/ml tetrahydroharmine, and 0.10 mg/ml harmaline. Participants ingested an average ± SD total volume of ayahuasca of 116.7 ± 17.1 ml, which contained the following amounts of alkaloids: 75.5 ± 11.1 mg DMT, 98.4 ± 14.4 mg harmine, 88.6 ± 13.0 mg tetrahydroharmine, and 11.3 ± 1.7 mg harmaline.

The ayahuasca tea used by group 2 contained the following alkaloid concentrations: 0.96 mg/ml DMT, 0.48 mg/ml harmine, 0.69 mg/ml tetrahydroharmine, and 0.10 mg/ml harmaline. Participants ingested an average ± SD total volume of ayahuasca of 44.5 ± 15.6 ml, which contained the following amounts of alkaloids: 42.8 ± 14.9 mg DMT, 21.4 ± 7.5 mg harmine, 30.8 ± 10.8 mg tetrahydroharmine, and 4.6 ± 1.6 mg harmaline.

Two parallel versions of the creativity tests were administered before and after ayahuasca intake. The order of administration was counterbalanced between participants, and the total time to complete the battery was around 35 min.

### Creativity tasks

Two verbal creativity tasks with non-verbal stimuli were used, i.e., the Pattern/Line Meanings task (PLMT) and the Picture Concept Task (PCT).

The PCT was composed of stimuli from the Wechsler Preschool and Primary Scale of Intelligence and the Wechsler Intelligence Scale for Children. Each stimulus contains between 4 and 12 color pictures shown in two or three rows. Participants have to find an association between one of the pictures in each row. They are instructed to provide the correct solution as there is only one correct answer. Correct answers are taken as the dependent measure of convergent thinking. In order to be also able to assess divergent thinking, we additionally asked participants to provide as many alternative answers as possible. This is the regular instruction included in measures of divergent thinking, and it is used to calculate several parameters, i.e., fluency, originality, and the ratio of both, which reflect quantity and quality of divergent thinking. Fluency is defined as the number of alternative associations. The second parameter, i.e., originality, is calculated by evaluating the originality of the alternative association relative to those provided by all other participants in a session. Alternative answers that were uniquely reported by a single participant received an originality score of 2. Answers that were shared with a single participant were valued as 1, and answers that were shared by 3 or more participants were rated zero. Mean originality (creativity) scores and ratio originality scores weighed for fluency (originality/fluency) were used as measures of divergent thinking. In total, 28 stimuli were shown and participants had 1 min per stimulus.

In the PLMT, the participant had to give meaning to a configuration of patterns or lines and generate as many explanations for it as possible, trying to be as creative as possible. In total, eight items were shown and participants were allowed 2 min for each pattern (Claridge and McDonald 2009). The total number of interpretable, meaningful, and relevant ideas generated in response to the stimulus (i.e., fluency) and the originality of these ideas were taken as dependent measures of divergent thinking. Originality and the ratio between originality and fluency were calculated as described in the description of the PCT.

### Subjective effects—visual analog scales

Once the acute effects had subsided, participants were asked to rate the intensity of various aspects of the acute ayahuasca experience using visual analog scales (VAS). These VAS were horizontal lines ranging from 0 (minimum) to 100 (maximum). VAS items included the following: “visual effects with eyes closed “visual effects with eyes open,” “bodily sensations,” “auditory phenomena,” “modifications of affect,” “autobiographic memories,” “emotional memories,” “detachment from own thoughts,” “confronted with past events,” “confronted with personal issues,” “overwhelmed by the experience,” “fear,” “increased closeness to others,” “increased detachment from others,” “experience of unity with universe,” “physical comfort,” “physical discomfort,” “self-acceptance,” “forgiving oneself,” “forgiving others,” “wellbeing,” “euphoria,” “sense of contact with god or a supernatural force,” “happiness,” “sadness,” “sense of rebirth,” “new intuitive revelations,” and “overall intensity of the experience.” Participants had to make a vertical mark on each line according to the intensity with which they had felt a given effect during the acute inebriation.

### Statistics

Data was analyzed by means of the statistical package IBM SPSS version 21. Independent sample *t* tests were conducted on sociodemographic measures to check for potential differences between groups. A mixed factorial general linear repeated measures model with “group” (1, 2) as between subject factor and “Ayahuasca” (before (baseline), after (ayahuasca ingestion)) as within subject factor was conducted.

Subjective effects induced by ayahuasca were analyzed separately for each group using one-sample *t* tests comparing the scores on each VAS after ayahuasca versus a zero distribution since previous studies have shown that placebo scores are typically very low, not rising above 0 in a scale from 0 to 100 (e.g., Valle et al. 2016). Additionally, potential differences in VAS scores between groups were analyzed using independent samples *t* tests.

The significance level for all analyses was set at *p* = .05.

## Results

### Sociodemographic variables

Independent sample *t* tests showed significant differences between groups on age (*t*_12.90_ = −3.47; *p* = .004) and years of education (*t*_21.43_ = −3.04; *p* = .006). With a mean difference of 14.6 years, participants in group 2 were older than those in group 1. Participants in group 2 had on average 2.9 years more education compared to group 2. The two groups did not differ in the number of times participants had used ayahuasca (*t*_10.70_ = −1.62; *p* = ns).

### Subjective effects—visual analog scales

**Fig. 1:**
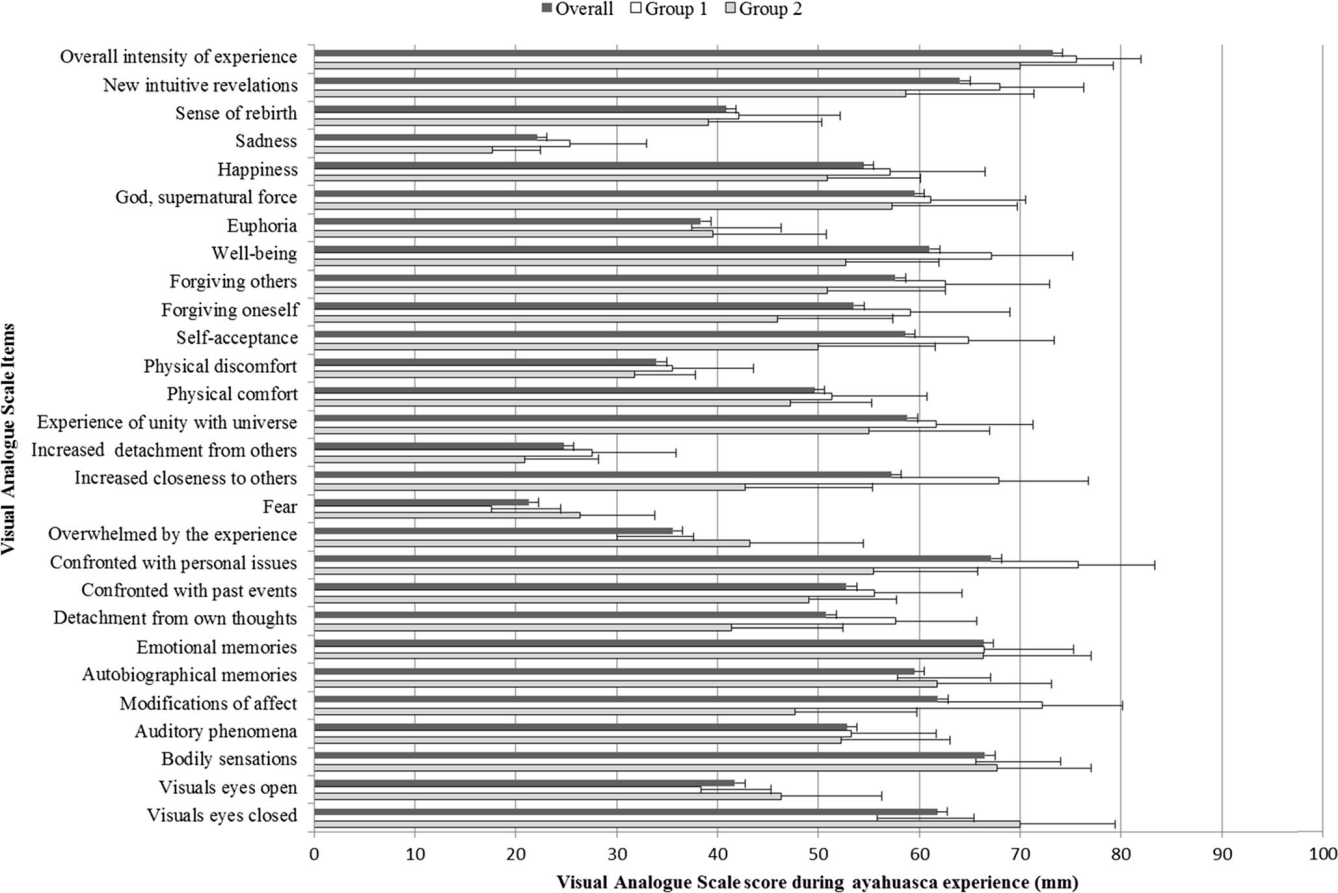
Mean (±SE) of visual analog scale scores on 28 items during the ayahuasca experience

Mean ± SD ratings on the different VAS items after ayahuasca are shown in Fig. 1 separately for each group. The one-sample *t* tests showed ayahuasca-induced significant increases in all VAS items in the two groups relative to 0, i.e., group 1 (*t*_14_ = 2.56–11.79; *p* ≤ .001–.023) and group 2 (*t*_10_ = 2.86–7.57; *p* ≤ .001–.017). Comparisons between groups did not find differences in mean scores for any of the VAS items (*F*_24_ = .003–1.77; *p* > .05).

### Divergent thinking

Analyses of PCT measures showed a significant main effect of group on fluency, originality, and ratio (originality/fluency). Fluency and originality scores were higher in group 1 compared to group 2 while ratio was higher in group 2 compared to group 1. PCT analyses also revealed an interaction effect of ayahuasca by group on fluency. The latter indicating that while fluency decreased in group 1 after ayahuasca ingestion compared to baseline performance, the opposite effect was seen in group 2, i.e., they improved under influence of ayahuasca. A main effect of ayahuasca on ratio was shown in the PCT demonstrating that when corrected for fluency, originality scores improved under influence of ayahuasca compared to baseline (see Table 1).

**Table 1:** Mean (±SE) and GLM outcomes of dependent variables of the creativity tasks per group and averaged over the two groups (“overall”)

	Task	Factor	Mean (±SE)						GLM					
		Ayahuasca	Before ayahuasca			After ayahuasca			Ayahuasca		Group		Ayahuasca*Group	
		Group	1	2	Overall	1	2	Overall	*F*	*p*	F	*p*	*F*	*p*
DT	PLMT	Fluency	38.53 (4.80)	44.18 (3.24)	41.36 (3.14)	41.60 (5.87)	41.45 (3.60)	41.53 (3.77)	.009	ns	.17	ns	2.61	ns
		Originality	55.73 (8.10)	63.64 (4.61)	59.68 (5.15)	57.00 (11.75)	62.00 (6.27)	59.50 (7.40)	.001	ns	.31	ns	.07	ns
		Ratio	1.41 (.05)	1.45 (.04)	1.43 (.03)	1.32 (.11)	1.50 (.07)	1.41 (.07)	.105	ns	1.47	ns	1.03	ns
	PCT	Fluency	40.67 (3.23)	16.54 (3.79)	28.61 (2.49)	34.47 (3.47)	19.45 (4.17)	26.96 (2.70)	.601	ns	17.06	<.001	4.61	.042
		Originality	43.60 (5.23)	21.45 (4.82)	32.53 (3.69)	42.07 (4.85)	28.91 (6.39)	35.49 (3.94)	.812	ns	6.56	.017	1.87	ns
		Ratio	1.04 (.05)	1.23 (.07)	1.14 (.04)	1.18 (.07)	1.42 (.10)	1.30 (.06)	5.902	.023	7.72	.010	.14	ns
CT	PCT	Correct	12.73 (.89)	15.27 (1.57)	14.00 (.85)	11.20 (1.03)	12.45 (1.70)	11.83 (.94)	6.624	.017	1.44	ns	.58	ns

*DT* divergent thinking, *CT* convergent thinking, *ns* not significant

There were no statistical significant effects of ayahuasca, group, or their interaction on the dependent measures of the PLMT.

### Convergent thinking

**Fig. 2:**
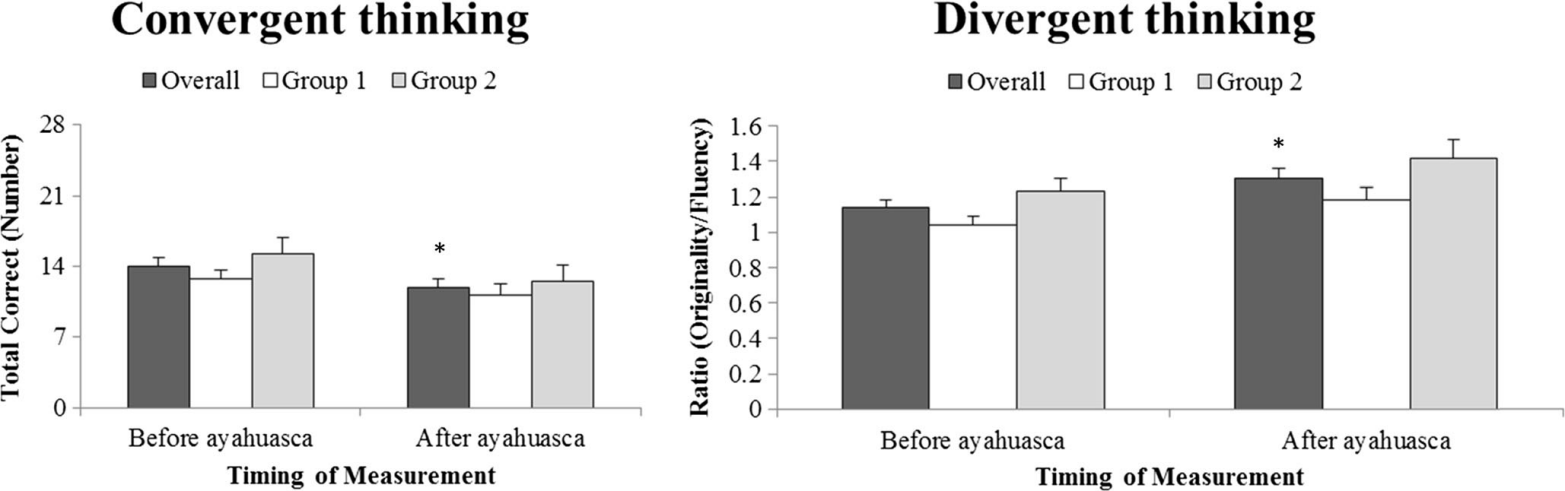
Convergent thinking (*left side*) and divergent thinking (*right side*) as measured with the PCT, before and after ayahuasca ingestion. The *white bar* represents the mean (±SE) of group 1, they *gray bar* of group 2, and the *dark bar* the mean of the two groups together. Convergent thinking deteriorated after ayahuasca ingestion while divergent thinking improved as indicated by the *asterisk* (*)

Statistical analysis showed a main effect of ayahuasca on the number of correct responses in the PCT indicating that convergent thinking decreased after ingestion of ayahuasca compared to baseline performance (see Table 1). There was no significant effect of group on the number of correct responses in the PCT (Fig 2).

## Discussion

The aim of the present study was to further investigate the psychological mechanisms underlying the ayahuasca experience, and specifically whether ayahuasca acutely affects creative thinking. It was shown that during the acute inebriation, ayahuasca caused a decrease in conventional convergent thinking and enhanced creative divergent thinking, as measured by the PCT. All dependent variables associated with the PLMT remained unaffected.

The ayahuasca-induced enhancement of divergent thinking could potentially be linked to the effects ayahuasca exerts on brain regions involved in creativity. Three core networks, i.e., the default mode network (DMN), including the ventromedial prefrontal cortex and posterior cingulate cortices, the central executive network (CEN), including dorsolateral prefrontal cortex and posterior parietal cortices, and the salience network (SN), including dorsal anterior cingulate cortex and anterior insula, interact during divergent thinking via corticostriatal-thalamocortical loops (Chávez-Eakle et al. 2007; de Manzano et al. 2010; Fink et al. 2009; Geyer and Vollenweider 2008; Jung et al. 2013; Pinho et al. 2015). The thalamus feeds information into the SN which in turn coordinates the other mentioned networks (Beaty et al. 2016; Uddin 2015). The SN monitors events occurring outside of the body as well as internal consciousness and is able to direct attention to whatever is more important at a certain moment in time. It is suggested that especially these shifts between these externally (CEN) and internally (DMN) oriented cognitive networks are very important in creative divergent thinking (Jung et al. 2013; Perlovsky and Levine 2012). Interestingly, previous research has shown that ayahuasca reduced thalamic gating of sensory and cognitive information (Riba et al. 2002). Taking into account the neuronal pathways previously described, this could lead to an increase in information fed into the salience network. Consistent with this line of thinking is the fact that Riba and colleagues (2006) showed an increase in blood perfusion in the SN after ayahuasca ingestion. Previously, other imaging studies with psychedelics also showed increased blood perfusion or a higher metabolic rate of glucose in the frontal and paralimbic areas during (e.g., anterior insula, anterior cingulate) psilocybin and mescaline (Gouzoulis-Mayfrank et al. 1999a, b; Hermle et al. 1992; Riba et al. 2006; Vollenweider et al. 1997). In addition, Alonso et al. (2015) found broad-band power decrements in the EEG signal after ayahuasca compared to placebo. Based on the knowledge about negative correlations between EEG and BOLD (Moosmann et al. 2003), this was interpreted as reflecting increased activation of areas involved in visual processing and in the cognitive-emotional processing anterior cingulate cortex (ACC), an area that is central to the SN.

Alonso et al. (2015) also showed a temporary induced disruption of neural hierarchies by ayahuasca, i.e., by reducing top-down control and increasing bottom-up information transfer in the human brain (Alonso et al. 2015). It was suggested that the higher excitability of posterior regions in combination with the loosening of the cognitive grip exerted by frontal regions responsible for executive control may underlie the associations and insights that emerge during the experience (Alonso et al. 2015). Palhano-Fontes and colleagues (2015) also found other parts of these networks to be influenced by ayahuasca, i.e., they showed a decrement in the functional connectivity in parts of the DMN after ayahuasca ingestion (Palhano-Fontes et al. 2015). It was suggested that this could result in more cognitive flexibility and consequently potentially enhanced divergent thinking (Carhart-Harris et al. 2014). However, Petri et al. (2014) suggest that the picture is more complex as they showed that the brain does not become a random system after psilocybin administration but still has stable connections which are different from the placebo state and only present in the psychedelic state (Petri et al. 2014). Likewise, Roseman et al. (2014) showed an increase in between-network resting state functional connectivity under psilocybin across normally distinct brain networks (Roseman et al. 2014). This increased integration between cortical areas could give rise to more associations (Petri et al. 2014) and an increased influence of imagination on visual perception (Roseman et al. 2014). Still, the precise neurobiological underpinning of how psychedelics may enhance divergent creative thinking remains largely unknown, and further research is warranted.

Convergent thinking can be seen as the second phase in the creative thinking process, i.e., focused on narrowing possibilities to a workable solution after the ideas have been generated through divergent thinking (Hennessey and Amabile 2009). Studies have shown this phase to be associated with an increase in CEN activity (Sowden et al. 2015). In the present study, ayahuasca caused deterioration in convergent thinking. Palhano-Fontes and colleagues (2015) found that ayahuasca only influenced activity in the DMN without changing the connection between DMN and CEN (Palhano-Fontes et al. 2015). The absence of ayahuasca enhancing effects on the CEN, together with the decrease in thalamic gating and loosened cognitive control described by Alonso and colleagues (2015), could explain the negative effect of ayahuasca on convergent thinking (Alonso et al. 2015). However, research with other psychedelics, e.g., psilocybin, showed an increase in functional connectivity between the DMN and the task-positive network or CEN (Carhart-Harris et al. 2013; Roseman et al. 2014).

Ayahuasca selectively affected performance in the PCT and not in the PLMT. Anecdotal reports from participants suggest that the stimuli of the PCT elicited more novel thoughts due to their more complex and colorful nature. In contrast, the stimuli in the PLMT contrast were very simple black-and-white line drawings. The latter probably gave less input into the system involved in the generation of new ideas. Another point to be mentioned is the quasi-experimental design of this study which potentially limits the conclusions that can be drawn from it. It could be argued that because ayahuasca sessions always followed the baseline session, and as it is known that ideas can get more creative over time (Beaty and Silvia 2012), a potential order effect could have influenced the results. However, besides the fact that parallel versions of tasks were used in a randomized order to counter potential order effects, the double dissociation, i.e., improvement of divergent thinking and impairment of convergent thinking, suggests that results were not subjected to the serial order effect.

Previously, it has been shown that the connection between divergent thinking and mood is particularly strong and positive (Baas et al. 2008; Davis 2009), i.e., more positive mood improves divergent thinking. In contrast, convergent thinking and mood are related in a negative way: more positive mood lowers convergent thinking. Mood of participants in the present study was generally very positive, which may have contributed to their openness to creative ideas. Mood ratings were only taken after drinking ayahuasca and could not be compared to mood states before drinking; nonetheless, they differed statistically from 0. In the future, placebo-controlled studies including measures of mood could test whether mood changes are a moderator in the effects of ayahuasca. Another point which could be addressed in future research is the usefulness of ideas generated in the divergent thinking task as this is part of the definition of divergent creative thinking. By including for example a task in which this quality can be assessed, e.g., the “alternate uses task,” it can be tested whether ayahuasca also improves this aspect of divergent thinking.

It would be interesting and important in the light of potential clinical applications of ayahuasca to investigate whether the effects are stable or also vary in time. It is known that ayahuasca induces an intense modified state of consciousness, starting between 35 and 40 min after administration and lasting approximately 4 h (Frecska et al. 2016). We showed that during this period, divergent thinking was enhanced and convergent thinking distorted. Soler et al. (2015) demonstrated that 24 h after ayahuasca intake, mindfulness-related capacities were enhanced (Soler et al. 2015). Mindfulness, a state of nonjudgmental, sustained, and alert awareness which improves people’s cognitive, emotional, and interpersonal functioning, has been shown to be linked with convergent thinking and less with divergent thinking (Lebuda et al. 2016). Based on these findings, it could be interesting to see whether convergent thinking is recovered 24 h after ayahuasca administration. It is suggested, based on a study of Bouso et al. (2008) on MDMA-assisted psychotherapy in post-traumatic stress disorder, that the potential effect pattern of ayahuasca would make it suited for psychedelic-assisted psychotherapy. The increase in divergent thinking during the acute phase could help patients relive events, recalling various associations without feeling inhibited (Bouso et al. 2008; Frecska et al. 2012, 2016). The sub-acute effects could then be suited in a second, “integration” session in which patients discuss the experiences they had on ayahuasca and find strategies that help them cope with intensive emotions. Future studies should therefore not only focus on either the acute or sub-acute phase but also take both stages into account.

In the past decade, a renewed interest in the therapeutic potential of psychedelics has emerged (Anderson 2012; McKenna 2004; Sessa and Johnson 2015). The present study has shown that ayahuasca promotes divergent thinking, an ability which has been shown to be an important aspect in cognitive therapy (Forgeard and Elstein 2014). It can therefore be suggested that ayahuasca possesses qualities that can promote a therapeutic process. However, since convergent thinking is also a critical aspect in therapy, and the current findings show that ayahuasca impairs this facet during the acute phase, future studies have to investigate whether this effect profile changes over time. Additional research utilizing a placebo-controlled experimental design, including additional creativity measures, is warranted, before results can be generalized.
